# Incomplete recovery of seeds from scatterhoards by granivorous rodents: Implications for plant establishment

**DOI:** 10.1002/ece3.8523

**Published:** 2022-01-13

**Authors:** Keith Geluso, Peter C. Longo, Mary J. Harner, Jeremy A. White

**Affiliations:** ^1^ Department of Biology University of Nebraska at Kearney Kearney Nebraska USA; ^2^ Department of Communication University of Nebraska at Kearney Kearney Nebraska USA; ^3^ Department of Biology University of Nebraska at Omaha Omaha Nebraska USA

**Keywords:** *Dipodomys ordii*, incomplete recovery, kangaroo rats, plant establishment, scatter hoarding

## Abstract

Granivorous rodents are important components of ecosystems not only because they consume seeds but also because some aid in seed dispersal through seed‐caching behaviors. Some rodents bury seeds in shallow pits throughout territories, called scatterhoards, that individuals recover, pilfer, or transfer to other caches. We suspect some single‐seed caches in environments represent missed seeds from reclaiming or pilfering caches. We documented the sloppiness of seed removal from scatterhoards of soapweed yucca (*Yucca glauca*) seeds by Ord's kangaroo rats (*Dipodomys ordii*). We quantified the frequency and location of seeds remaining. In an experiment with artificial caches of three sizes, kangaroo rats harvested 51% of caches after one night, and 53% had incomplete recovery with at least one seed remaining. The greater the number of seeds in caches, the greater frequency of incomplete recovery. In another experiment with natural and artificial caches, 75% of caches were excavated after 8 days, with at least 70% having at least one seed remaining. Regardless of original cache size, a single seed represented the mode for seeds remaining. Incomplete recovery of seeds likely benefits plant establishment, potentially significantly in some systems. Remaining seeds, especially those buried at bottoms of caches, likely will stay undetected in landscapes, yielding propagules for subsequent plant generations. Soapweed yucca has large but light, flat wind‐dispersed seeds, and removal of caches with smaller seeds might have greater frequency of missed seeds during recovery and pilfering by rodents. Our results suggest that scatter‐hoarding granivores also contribute to plant establishment by leaving limited numbers of seeds behind when removing caches, at least in some systems.

## Graphical abstract

We documented the sloppiness of seed removal from scatterhoards of soapweed yucca (*Yucca glauca*) seeds by Ord's kangaroo rats (*Dipodomys ordii*). In two different experiments, we observed that >50% of caches removed or pilfered by kangaroo rats in the Sandhill Region of Nebraska had at least one seed remaining, with about 30% leaving limited seeds at the bottom of caches. Remaining seeds, especially those buried at bottoms of caches, likely will stay undetected in landscapes, yielding propagules for subsequent plant generations.

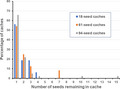

## 1 INTRODUCTION

Granivorous rodents are important components of ecosystems not only because they consume many plant seeds but also because some species aid in seed dispersal and plant propagation via seed‐caching behaviors (Price & Jenkins, 1986; Vander Wall, 1990, 1993). Unlike larder‐hoarding rodents that generally deposit food resources in a single, centrally located cache deep inside burrows, scatter‐hoarding rodents bury many small quantities of seeds at shallow depths throughout territories (Reynolds, 1958; Stapanian & Smith, 1978; Vander Wall, 1990, 1993). Scatter‐hoarding individuals can have hundreds or thousands of caches, each containing many seeds (Shaw, 1934; Vander Wall & Jenkins, 2003; Vander Wall & Joyner, 1998). In fact, Vander Wall (2019) noted that potentially thousands of seeds are harvested each season by each scatter hoarder in a population. Scatter‐hoarding animals not only disperse seeds from masting plants but bury propagules at shallow depths in soils (McAdoo et al., 1983; Reynolds & Glendening, 1949; Vander Wall, 1993). Many seeds require burial to germinate, and Vander Wall (1993) further demonstrated that scatter‐hoarding rodents sometimes fortuitously bury seeds at optimal depths for germination, although any shallow depth below the soil surface is potentially advantageous. Each year some caches are not retrieved and germinate in spring or after periods of rain, erupting as clumped groups of seedlings from unharvested caches (Price & Jenkins, 1986; Roth & Vander Wall, 2005; Shaw, 1934; Vander Wall, 1994b). In arid and semi‐arid environments across western North America, scatter‐hoarding rodents are associated with the establishment of several valuable or conspicuous plant species (Dimitri et al., 2017; La Tourrette et al., 1971; Longland, 1995; Longland & Dimitri, 2019; McAdoo et al., 1983; McAuliffe, 1990; Reynolds & Glendening, 1949; Roth & Vander Wall, 2005; Vander Wall et al., 2006; West, 1968). In the Sierra Nevadas, for example, yellow pine chipmunks (*Tamias amoenus*) were shown to originally disperse and bury about 99% of emerging seedlings of antelope bitterbrush (*Purshia tridentata*) via scatter hoarding (Vander Wall, 1994b). Regeneration of antelope bitterbrush, as well as other plant species, including grasses, shrubs, and trees, appears to depend on seed‐caching rodents (Longland & Dimitri, 2019; Vander Wall, 1994b).

Besides burying seeds in primary caches, scatter‐hoarding rodents recache scatterhoards as well as pilfer and cache scatterhoards of conspecifics and other species (Dittel et al., 2017; Roth & Vander Wall, 2005; Vander Wall, 2002; Vander Wall, Esque, et al., 2006; Vander Wall & Jenkins, 2003; Vander Wall & Joyner, 1998). In fact, secondary or subsequent caches have larger effects on dispersal and germination of Jeffrey pine (*Pinus jeffreyi*) and bush chinquapin (*Chrysolepis sempervirens*) than primary caches (Roth & Vander Wall, 2005; Vander Wall & Joyner, 1998). Rodents not only rapidly sequester and cache seeds near masting plants but they also subsequently recache these food resources once hidden from competing granivores (Jenkins et al., 1995). Fluidity of seed movement due to caching, pilfering, and recaching by scatterhoarders has a significant effect on plant recruitment and establishment, with cascading population‐level and community‐level effects for producers (Longland, 1995; Longland et al., 2001; Vander Wall & Joyner, 1998).

**FIGURE 1:**
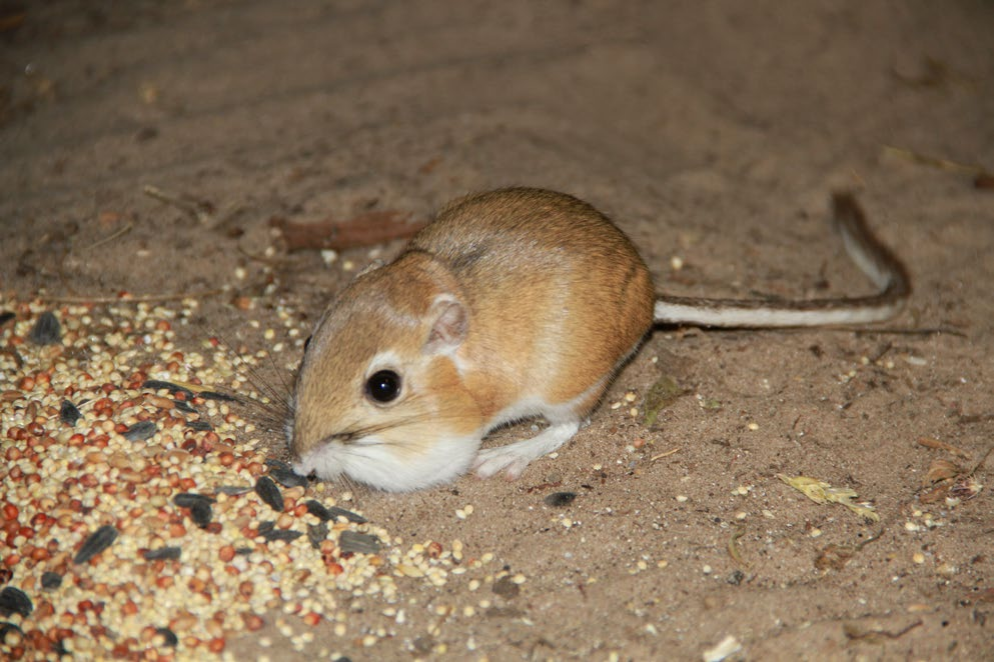
An Ord's kangaroo rat (*Dipodomys ordii*) from the Sandhills Region of Nebraska, USA, that demonstrates the species’ large hind feet and long tail for bipedal locomotion as well as large external fur‐lined cheek pouches used to carry seeds throughout territories. Photo by K. Geluso

Continuous caching and recaching of scatterhoards yields opportunities for missed seeds. Ancillary evidence supports that such behaviors sometimes result in an incomplete recovery of seeds from caches (Geluso, 2005; Jenkins et al., 1995; Tomback et al., 2005; Vander Wall, 1994b; Vander Wall et al., 2019). We suspect that some single‐seed caches observed in environments represent missed seeds from reclaiming or pilfering of caches rather than intentionally cached seeds. With artificial and natural caches of soapweed yucca (*Yucca glauca*) seeds, a species highly sought after by rodents at the site, we quantified the frequency and location of seeds remaining (i.e., incomplete recovery) in scatterhoards when pilfered or recovered by Ord's kangaroo rats (*Dipodomys ordii*) in semi‐arid habitats of western Nebraska (Figure 1). We hypothesized that incomplete recovery of seeds by *D*. *ordii* would increase with greater numbers of seeds in caches. We further discuss the potential for subsequent plant establishment with seeds remaining in or near cache sites. Understanding the fate of all seeds when caches are recovered or pilfered by seed‐caching rodents has important implications for plant establishment in arid and semi‐arid environments.

## 2 MATERIALS AND METHODS

We conducted experiments at Crescent Lake National Wildlife Refuge, Garden County, Nebraska. The refuge is in the Sandhill Region of the state and characterized by continuous rolling sand dunes covered by native grasses and forbs (Bleed & Flowerday, 1998). This grassland consists of mixed‐grass prairie used mainly for grazing by domestic livestock with limited row‐crop agriculture. Conspicuous vegetation in upland dune habitats included sand bluestem (*Andropogon hallii*), prairie sandreed (*Calamovilfa longifolia*), needle and thread (*Stipa comata*), sand muhly (*Muhlenbergia arenicola*), sand dropseed (*Sporobolus cryptanthus*), sunflowers (*Helianthus*), bractless blazingstar (*Mentzelia nuda stricta*), sand cherry (*Prunus pumila*), and soapweed yucca. Climate of the Sandhill Region is characterized by cold winters (average minimum temperature for January was −12.9°C) and warm summers (average maximum temperature for July was 30.7°C), and >80% of precipitation falls April–September (Wilhite & Hubbard, 1989).

We conducted two experiments to examine the incomplete recovery of caches by Ord's kangaroo rats. Experiment 1 used artificial caches of three discrete sizes, whereas Experiment 2 examined naturally deposited caches and nearby artificial caches with the same number of seeds. To provide a local source of seeds for experiments, we collected seeds of *Y*. *glauca* at the refuge by breaking apart ripe dehiscing pods. We sorted viable seeds from non‐viable seeds and wore vinyl gloves to prevent human scent from influencing detectability of seeds by rodents (Duncan et al., 2002; Wenny, 2002). Viable seeds have dark black coloration and presence of an endosperm identified by a centrally located, circular protrusion that slightly raised the seed coat, whereas unviable seeds have pale‐colored seed coats and lack endosperm (Addicott, 1986).

In September 2007, we conducted Experiment 1 with artificial caches that consisted of 18, 61, and 94 seeds based on the range of seeds deposited by *D*. *ordii* in preliminary investigations at the study site (2–110 seeds; J.A. White unpublished data). Caches with 18 seeds represented about 15% of the maximum cache size, 61‐seed caches represented the mean, and 94‐seed caches represented 85% of maximum cache size. Seeds were stored in individually labelled sterile polyethylene Whirl‐Pak bags (Nasco Company, Fort Atkinson, WI) prior to use at cache sites. We conducted Experiment 1 along sandy four‐wheel drive roads near refuge headquarters between Goose and Gimlet lakes (41.769312°N, 102.443069°W) in habitats frequented by kangaroo rats. Burrows and associated trails of kangaroo rats were abundant in the area. Artificial caches were placed within about 5 m of the road. We made triangular imprints with a 30.5 cm ruler in sandy areas lacking vegetative cover. At each corner, we placed one of each of the three sizes of artificial caches. Each cache in the group of three caches was located far enough away from the other two caches that we were able to discern which original cache any remaining seeds were from, based on our prior experiences of how kangaroo rats remove seeds from such caches. We intentionally placed the three caches close together in a group to potentially increase removal by individuals, if they could detect them, to increase our sample size of pilfered caches. Wearing vinyl gloves, we excavated a small shallow pit to a depth of about 15 mm, the approximate depth of caches made by Ord's kangaroo rats at the study site (J.A. White, unpublished data). At each site, we created an 18‐seed cache, a 61‐seed cache, and a 94‐seed cache, and we covered caches with excavated sand. Next, we moved a minimum of 25 m along the road to deploy another set of three caches to increase the opportunity to place caches in home ranges of multiple kangaroo rats. We rotated the orientation of the caches one position clockwise at each subsequent site. We placed caches at 70 sites for a total of 210 caches for this experiment.

In August 2008, we conducted Experiment 2 with natural and artificial caches. To determine the location of natural caches of Ord's kangaroo rats, footprints were followed after individuals collected yucca seeds from trays covered in powdered fluorescent pigments (Lemen & Freeman, 1985; Longland & Clements, 1995). Trays consisted of a cookie sheet lined with sandpaper and a Petri dish affixed to the center with 10 g of yucca seeds dusted with either 1 g of yellow or orange fluorescent powder (White & Geluso, 2012). Sandpaper was covered with the other color of fluorescent powder, which aided in finding seed caches among footprints. Sites with trays were ≥100 m apart to prevent the same kangaroo rat from collecting seeds at multiple trays. If seed caches were discovered, we uncovered natural caches and recorded number and depth to top of seeds. Powdered seeds were replaced with the same number of unpowdered yucca seeds to the same depth and covered with sand. Two similar sized artificial caches were created at the same depth and in the same microhabitat (i.e., surrounded by dense vegetation, edge of cover, or open) as the original cache, one about 30 cm and another about 100 cm away from the original cache. Those caches were associated originally with another project examining removal of caches by naive foragers compared to individuals that cached the seeds. Distance of 30 cm was replicated from a similar study by Vander Wall, Briggs, et al. (2006), whereas the distance of 100 cm was selected arbitrarily. A small twig was placed upright in the sand nearby to aid in locating caches.

The following mornings, we recorded the number of caches pilfered by *D*. *ordii*. All caches were examined and removed after one night for Experiment 1, whereas caches were monitored for removal for 8 days in Experiment 2. For Experiment 2, we combined the two types of artificial caches, and for original caches made by kangaroo rats, we separated those data from artificial caches as individuals potentially left seeds behind intentionally (see Vander Wall, 1995). Ord's kangaroo rats leave characteristic tail drags in sand and diagnostic foot imprints that allowed us to identify whether cache sites were visited by *D*. *ordii* (Geluso, 2005). Ord's kangaroo rat is the only species of *Dipodomys* in the region, with smaller rodents as the only other nocturnal granivores in the study area (Jones, 1964). Only caches visited by Ord's kangaroo rats were reported hereafter. At cache sites, we sifted sand for seeds and noted the number and placement of remaining seeds at excavated caches. We quantified seeds remaining into three categories based on location, (1) seeds remaining buried near the bottom of original cache sites (hereafter = buried), (2) those displaced from the cache pits but exposed aboveground (hereafter = exposed), and (3) those displaced from the cache and covered with excavated sand (hereafter = covered). In Experiment 1, Chi‐square statistics were used to determine whether pilferage differed based on cache size, as well as whether frequency of seeds remaining at cache sites differed based on cache size and placement (i.e., whether seeds remaining after incomplete recovery were buried, exposed, or covered). In Experiment 2, we used a Chi‐square statistic to examine whether frequency of seeds remaining differed among original and artificial (close or far) caches and whether frequency of seeds left behind at artificial caches differed based on cache placement.

## 3 RESULTS

In Experiment 1, kangaroo rats pilfered 51.4% (108 of 210) of total artificial caches of three discrete sizes after the first night. Individuals pilfered 34 (48.6%) of the 18‐seed caches, 36 (51.4%) of the 61‐seed caches, and 38 (54.3%) of the 94‐seed caches, with no difference detected in pilferage between cache sizes (*χ* ^2^ = 0.2, df = 2, *p* = .9). We documented the incomplete recovery of seeds at 52.8% (*n* = 57) of pilfered cache sites. The greater the original number of seeds in caches, the greater the number of caches with at least one seed left behind, as 18‐seed caches had 35.3% with at least one seed remaining, 61‐seed caches had 52.8%, and 94‐seed caches had 68.4%. Smaller caches more often were harvested completely, with no seeds remaining, than larger caches (*χ* ^2^ = 26.5, df = 2, *p* < .0001; 64.7% for 18‐seed caches with 28 total seeds remaining, 47.2% for 61‐seed caches with 48 total seed remaining, and 31.6% for 94‐seed caches with 80 total seeds remaining).

**FIGURE 2:**
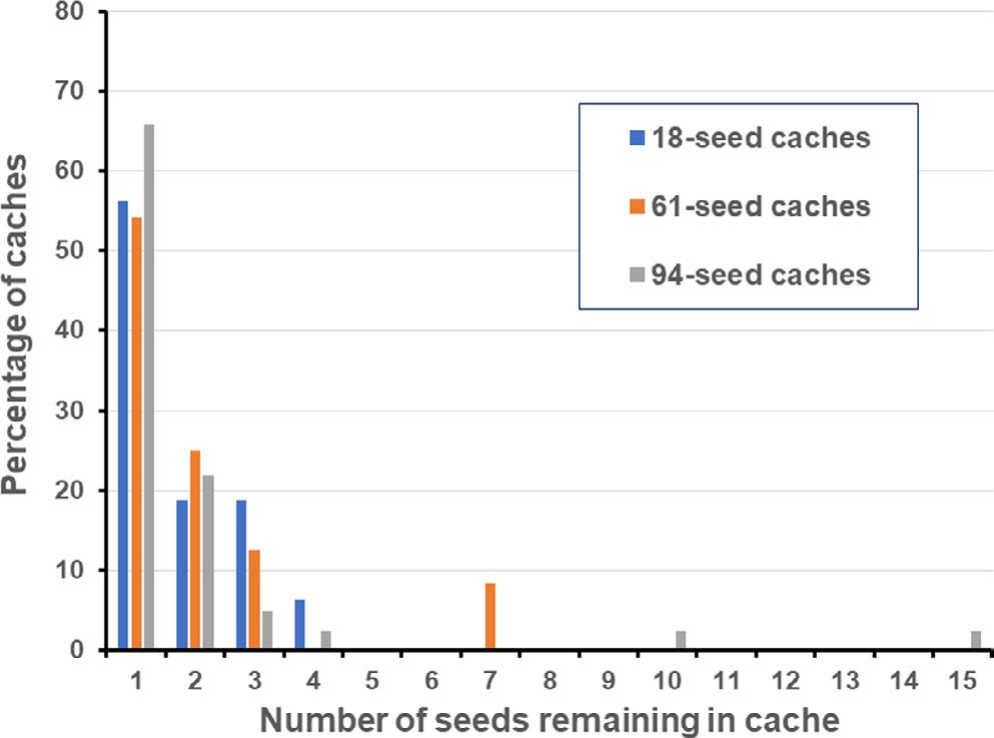
Percentage of caches with soapweed yucca (*Yucca glauca*) seeds remaining in or near caches after pilferage by Ord's kangaroo rats (*Dipodomys ordii*) from artificial caches containing different numbers of original seeds in the Sandhill Region of Nebraska, USA

Of pilfered caches with at least one seed remaining, 29.6% had buried seeds in the original pit, 30.6% had excavated exposed seeds, and 14.8% had excavated but covered seeds (Table 1). About 20% of caches with incomplete recovery had seeds remaining in multiple locations associated with caches. Although caches with greater numbers of original seeds had the greatest rates of incomplete recovery, the median and mode for seeds remaining at those different locations was a single seed, regardless of original cache size (Figure 2). The average number of seeds remaining per cache was about two seeds due to a few outlier caches with as many as 15 seeds remaining at one of the large caches. We detected a significant difference among the total number of remaining seeds at the different locations, as 72 seeds remained buried at the bottom of pits associated with originally cached seeds, 54 seeds were excavated and exposed near cache sites, and 30 seeds were excavated and covered with sand near cache sites (*χ* ^2^ = 17.1, df = 2, *p* = .0002; Table 1).

**TABLE 1:** Total number, average, and range of yucca seeds (*Yucca glauca*) remaining at cache sites as a result of incomplete recovery during pilfering by Ord's kangaroo rats (*Dipodomys ordii*) in the Sandhills of Nebraska

Placement and cache size	Total seeds remaining	Average seeds remaining	Range of seeds remaining
Buried seeds (total)	72	0.67	1–15
18	9	0.26	1–3
61	14	0.39	1–3
94	49	1.29	1–15
Exposed seeds (total)	54	0.50	1–7
18	13	0.38	1–4
61	23	0.64	1–7
94	18	0.47	1–2
Covered seeds	30	0.28	1–7
18	6	0.18	1–3
61	11	0.31	1–7
94	13	0.34	1–3

Note

Average number of seeds remaining was calculated from the total number of caches pilfered for that size of cache (18‐seed caches, *n* = 34; 61‐seed caches, *n* = 36; and 94‐seed caches, *n* = 38) or for totals for each placement out of total caches pilfered (*n* = 108).

In Experiment 2, we tracked individuals to a total of 30 original caches at 11 different sites, with caches averaging 49.9 seeds that ranged in size from 14 to 74 seeds. A total of 74.4% (67 of 90) of caches were removed or pilfered by kangaroo rats by day 8, including 20 close artificial caches, 23 far artificial caches, and 24 original caches deposited by individuals. All three types of caches (close artificial, far artificial, and original) had about the same percentage of caches with at least one seed remaining (80.0% close, 87.0% of far, and 79.2% of original). However, the total numbers of seeds remaining differed among the three types of caches, with more seeds remaining at original caches (85 seeds) than close artificial (52 seeds) and far artificial (54 seeds; *χ* ^2^ = 10.3, df = 2, *p* = .006).

In Experiment 2 for artificial caches with at least one seed remaining (*n* = 36), 33.3% had buried seeds in the original pit, 75% had excavated exposed seeds, and 36.1% had excavated but covered seeds. About 42% of caches with incomplete recovery had seeds remaining in multiple locations. Similar quantities of seeds remained covered (20 seeds) and buried (22 seeds), whereas many more remained exposed on the surface (64 seeds) in this experiment, with a difference observed between these three locations (*χ* ^2^ = 33.7, df = 2, *p* < .0001).

In Experiment 2 for original caches with at least one seed remaining (*n* = 19), 47% had buried seeds in the original pit, 68% had excavated exposed seeds, and 52% had excavated but covered seeds. About 58% of caches with incomplete recovery had seeds remaining in multiple locations. We observed no difference in the number of seeds remaining at the three locations (covered = 20 seeds, buried = 30 seeds, and exposed = 35; *χ* ^2^ = 4.1, df = 2, *p* = .13). The original cache with the largest number of seeds (74 original seeds) had 13 seeds remaining at the bottom of the pit when removed or pilfered, with three other larger caches (52, 65, and 66 seeds) having four seeds remaining at the bottom of the pit. Of the 36 artificial caches in Experiment 2 with at least one seed remaining, the largest number of seeds remaining at the bottom of a pit was 4 from a cache that originally contained 61 seeds.

## 4 DISCUSSION

Our study documented the incomplete recovery of seeds during pilferage of artificial scatterhoards by Ord's kangaroo rats. Few prior studies specifically present actual observations on this behavioral aspect of pilfering or reclaiming caches by granivorous rodents (Geluso, 2005; Jenkins et al., 1995), although other studies mention possible examples of incomplete recovery (Tomback et al., 2005; Vander Wall, 1994b, 1995, 2019). Jenkins et al. (1995) observed that Merriam's kangaroo rats (*Dipodomys merriami*) sometimes left 1–3 sunflower seeds when reclaiming their own caches in laboratory arenas. Geluso (2005) noted that when artificial caches were removed by kangaroo rats in a field situation that 1 or 2 seeds occasionally were left or missed when pilfering millet seeds. Rodents in eastern Colorado sometimes (53%) left 1 or 2 limber pine (*Pinus flexilis*) seeds at cache sites after visiting scatterhoards, but it was unclear whether this behavior was accidental or intentional (Tomback et al., 2005). Vander Wall (1994) reported that 5% of antelope bitterbrush caches contained a single seed. However, we suspect some of those singletons might actually represent seeds missed during recaching or pilfering events by yellow pine chipmunks. Vander Wall (1995) reported that 47.5% of primary antelope bitterbrush caches were subsequently visited with only some seeds eaten and removed, potentially some representing incomplete recovery, but Vander Wall commented that seeds remaining were intentionally left and not simply overlooked. We find of note that in original caches made by a kangaroo rat in Experiment 2 of our study, the largest cache removed had 13 seeds remaining at the bottom of the pit and 3 other original caches had 4 seeds remaining. In our same experiment (Experiment 2) with almost twice the number of artificial caches, the greatest number of seeds remaining at the bottom of any pit from pilfered caches was four seeds from a single cache, suggesting that individuals retrieving their own caches might intentionally leave some seeds behind. In another study, Vander Wall et al. (2019) demonstrated that seed value perceived by pilfering rodents affected how many seeds were left remaining in pilfered caches. Thus, although it appears soapweed yucca seeds are highly sought after in certain seasons at our study site, it is unclear how perceived seed value may relate to seeds remaining after incomplete recovery of caches.

In our study, we documented >50% of pilfered caches had seeds remaining, with about 30% of those caches with at least one seed still buried in the original cache depression associated with removal by kangaroo rats. Additionally, the mode for seeds remaining at the different locations (buried, exposed, or covered) was a single seed for both experiments (Table 1). In general, prior studies demonstrate that caches with relatively few seeds are less vulnerable to detection by granivorous rodents across environments, especially associated with olfactory cues (Evans et al., 1983; Geluso, 2005; Reichman & Oberstein, 1977; Vander Wall et al., 2003). Single antelope bitterbrush seeds cached at a depth of 1 cm were not bothered by rodents, whereas 75% of caches containing two seeds were removed (Evans et al., 1983). Thus, when only single seeds, or limited numbers of seeds, are left behind at caches, granivorous organisms likely will not detect and harvest such seeds, increasing their longevity in the environment and yielding greater opportunities for such propagules to germinate and establish new plants. Additionally, once rodents remove or pilfer a cache, it seems there is a reduced likelihood that individuals will return to the cache site, again yielding a seed or a few seeds a better chance to persist in the environment.

Many environmental factors affect rates of harvesting and recovery of seeds (Price & Jenkins, 1986; Vander Wall, 1990; Vander Wall et al., 2019). Some of these factors also likely influence rates of incomplete recovery. Seed size and shape vary between plant species (Reichman, 1976; Smigel & Rosenzweig, 1974) as well as how rodents value different seed traits (Dimitri et al., 2017; Vander Wall et al., 2019). Smaller seeds as well as those seeds close in size and resembling substrate particles reduce or inhibit recovery by rodents (Price & Podolsky, 1989), and thus have the greatest likelihood of being mishandled or overlooked. In contrast, large or unusually shaped seeds relative to substrate particles likely will be recovered more efficiently, as heteromyid rodents appear to rely in part on tactile cues to extract and differentiate seeds from substrate and non‐edible organic particles (Lawhon & Hafner, 1981; Price & Podolsky, 1989; Reichman & Price, 1993). Other factors, such as olfactory, loading, and handling abilities of various rodents as well as frequency of rainfall events and attractiveness of seed composition (i.e., relative carbohydrate‐to‐protein ratios), also will influence incomplete recovery. Future studies are needed to examine the frequency and fate of such seeds across arid ecosystems, as we suspect incomplete cache recovery occurs more frequently than known, at least in some ecosystems and possibly more often with kangaroo rats.

Soapweed yucca seeds are relatively large compared to the sandy substrate at our site, which suggests few seeds should be left behind. Our relatively high rate of incomplete recovery with this large, distinctly shaped seed, especially for larger caches, might reflect the large number of seeds used in artificial caches. We do not know actual cache sizes made by kangaroo rats while harvesting natural quantities of seeds from yucca pods. Our large number of seeds was based upon kangaroo rats harvesting, and subsequently caching, seeds from artificially placed bait stations with abnormally large quantities of concentrated seeds (White & Geluso, 2012, J.A. White unpublished data). Larger caches have more seeds, and thus, a greater probability of rodents overlooking or mishandling seeds upon recovery. When kangaroo rats pilfered larger caches, it appeared individuals accidently kicked out seeds with sand while excavating caches, with some seeds covered by sand. In general, kangaroo rats can carry and cache more seeds in each scatterhoard than do chipmunks and deer mice (Vander Wall & Longland, 1999; Vander Wall et al., 1998, 2001), likely due to the larger capacity of their external fur‐lined check pouches, but this also relates to size of seeds and nuts harvested. Thus, those species that cache more seeds in scatterhoards might be more prone to incomplete recovery. Plus, different species or genera might be more prone to sloppiness when removing seeds from caches.

Pilferage rate of larger caches was not greater than smaller caches in this study. We predicted that smaller caches would be detected less frequently than larger caches, as cache size affects detection with larger caches being detected more frequently (Geluso, 2005; Reichman & Oberstein, 1977; Vander Wall et al., 2003). Potentially the size of our smallest caches with yucca seeds was above the threshold size for detection (Geluso, 2005), with still smaller caches needed to prevent olfactory detection. Another explanation was that the three caches at a site were too close together and once kangaroo rats detected one cache, the larger ones, individuals proceeded to search nearby and pilfer others. However, we suspect another clue was responsible for high pilferage rates of all caches, as soils were dry and crusted over for this experiment. Likely, some other aspect of our presence at artificial cache sites cued kangaroo rats to use exploratory digging to detect and recover artificial caches. We suspect our disturbance of soil compactness initiated exploratory digging by kangaroo rats, as moderately frequent summer rains create a crust‐like top layer when it dries that subsequently breaks down upon disturbance, such as walking and kneeling. Hall (1946) reported that dragging a boot heel in soil attracts heteromyids to traps, yet heteromyids including kangaroo rats did not respond to microtopographic features of the soil surface in a laboratory arena (Reichman & Oberstein, 1977).

The final location for remaining seeds with respect to original caches likely will have consequences for detection by granivores and for plant propagation. When a single or limited number of seeds remain buried at or near bottoms of original caches, such seeds are the least likely to be discovered by subsequently foraging granivores and persist longer in the environment (Evans et al., 1983; Vander Wall, 1994a). As mentioned previously, limited numbers of seeds buried in soils are harder to detect than larger numbers, even if seeds imbibe water and emit detectable organic solutes (Geluso, 2005; Reichman & Oberstein, 1977; Vander Wall, 1993, 2000; Vander Wall et al., 2003). Even if a forager detects buried seeds, it also may not attempt to retrieve them if the number of seeds is too small or the depth too great to repay the effort (Lockard & Lockard, 1971).

Incomplete recovery of caches likely is important for seed dispersal and establishment of soapweed yucca. Yucca seeds are described as wind dispersed (Dodd & Linhart, 1994), as these thin, light seeds can be carried by wind after dehiscent pods open. However, seeds of soapweed yucca are readily gathered and dispersed by kangaroo rats (White & Geluso, 2012), as we have also observed evidence of kangaroo rats chewing through pods to extract seeds. Vander Wall (1994a) demonstrated the importance of rodents, mainly chipmunks, in seed dispersal and germination of wind‐dispersed seeds for various pine species in the Sierra Nevadas. Thus, kangaroo rats also likely play a significant role in dispersal of soapweed yucca and other plants at our study site, even for those dispersed by other mechanisms.

The incomplete recovery of seeds by granivorous rodents represents another facet of plant establishment associated with scatter‐hoarding rodents previously not quantified in seed fate models. However, such seeds will be difficult to recognize in environments, as these single or limited seeds will appear established via non‐rodent‐mediated behaviors, unlike clumped seedlings erupting from non‐harvested scatterhoards. The few seeds remaining at cache sites appear ideal for plant propagation in some of the final locations where they remain. With the potential for high rates of pilfering and recaching of cached seeds (i.e., reciprocal pilfering hypothesis) in some systems (Vander Wall, 2000; Vander Wall & Jenkins, 2003, this study), our data suggest that a large number of single or limited seeds likely remain and influence plant recruitment and establishment in some arid and semi‐arid ecosystems.

## CONFLICT OF INTEREST

None declared.

## AUTHOR CONTRIBUTIONS

**Keith Geluso:** Conceptualization (equal); formal analysis (lead); investigation (equal); writing – original draft (lead); writing – review and editing (supporting). **Peter Longo:** Conceptualization (equal); formal analysis (supporting); investigation (equal); writing – original draft (supporting). **Mary Harner:** Conceptualization (equal); investigation (equal); writing – review and editing (equal). **Jeremy White:** Conceptualization (equal); investigation (equal); writing – review and editing (equal).

## Data Availability

Our data file has been uploaded to DRYAD (https://doi.org/10.5061/dryad.v9s4mw6xh).
